# The mediating role of sleep quality in the association between nocturia and health-related quality of life

**DOI:** 10.1186/s12955-019-1251-5

**Published:** 2019-12-11

**Authors:** Edmond Pui Hang Choi, Eric Yuk Fai Wan, Jojo Yan Yan Kwok, Weng Yee Chin, Cindy Lo Kuen Lam

**Affiliations:** 1 School of Nursing, University of Hong Kong, 4/F, William M.W. Mong Block, 21 Sassoon Road, Pokfulam, Hong Kong; 2 Department of Family Medicine and Primary Care, University of Hong Kong, Pokfulam, Hong Kong; 3 Department of Pharmacology and Pharmacy, University of Hong Kong, Pokfulam, Hong Kong

**Keywords:** Health-related quality of life, Mediation, Nocturia, Sleep quality

## Abstract

**Background:**

Even though the negative impacts of nocturia on sleep quality and health-related quality of life (HRQOL) have been documented in previous research, their interrelationship has been poorly studied. This study aimed to explore whether nocturia would affect sleep quality, which in turn affects HRQOL.

**Methods:**

Participants aged 40 and above were randomly recruited from a Hong Kong public primary care clinic. Participants were asked to report the average number of nocturia (waking up at night to void) pisodes per night over a 1-month period. The Pittsburgh Sleep Quality Index (PSQI) and the 12-Item Short Form Health Survey version 2 (SF-12 v2) were administered. The mediation analysis was tested using multistage regression approach and bootstrap method.

**Results:**

Of the 500 subjects who completed the survey, 31.2% reported symptomatic nocturia (having ≥2 nocturia episodes per night), and 60.4% experienced poor sleep quality (a PSQI global score > 5). Respondents with symptomatic nocturia had a poorer HRQOL in the domains of physical functioning (PF), role physical (RP) and social functioning (SF), general health (GH), vitality (VT) and physical component summary (PCS) of the SF-12 v2 than those without. Compared with the respondents without poor sleep quality, those with poor sleep quality had poorer HRQOL across all domains and summaries of the SF-12 v2. Mediation analysis found that sleep quality fully mediated the association between nocturia and the PF, RP and SF domains of the SF-12 v2, respectively, and partially mediated the association between nocturia and the GH, VT and PCS domains of the SF-12 v2, respectively.

**Conclusions:**

We found that sleep quality mediated the association between nocturia and HRQOL. To enhance the HRQOL of patients with nocturia, clinicians should not only focus on nocturia symptoms, but also on their sleep quality.

## Background

Nocturia is the most common among lower urinary tract symptoms [1]. An epidemiological study conducted in the United States (US), the United Kingdom (UK) and Sweden found that among people aged ≥ 40 years, the prevalence of having ≥ 2 nocturia episodes per night was 28% for men and 34% for women [2]. A study in Asia also found that the prevalence of having ≥2 nocturia episodes per night was 36% among people aged ≥ 40 years [3].

The impact of nocturia is not trivial. Studies in the US [4] and Sweden [5] also found that nocturia negatively affects health-related quality of life (HRQOL) as measured by the Short Form-36 Health Survey (SF-36). Notwithstanding, poor sleep quality is a common complaint among people with nocturia [6]. A study in the US reported that poor sleep quality can have a serious impact on the ability to function and daytime functioning, leading to impaired HRQOL [6, 7].

Even though the negative impacts of nocturia on sleep quality and HRQOL have been documented in previous research [6], their interrelationship has been poorly studied. In fact, a study of a Dutch population found that the impacts of nocturia on HRQOL became statistically insignificant after controlling for sleep quality. Besides, in a qualitative study in the US, respondents indicated that nocturia affected their functioning and wellbeing in daytime because of poor sleep quality [8]. These studies imply that the relationship between nocturia and HRQOL could be mediated by sleep quality. A better understanding of their interrelationship could help clinicians better manage patients with nocturia by informing the development of interventions to address their multidimensional needs.

Therefore, the objective of the present study was to explore the interrelationship between nocturia, sleep quality and HRQOL. We hypothesized that sleep quality mediated the relationship between nocturia and HRQOL.

## Methods

### Study design

This was a cross-sectional study.

### Setting

The study participants were recruited from a government-funded primary care clinic of the Hospital Authority, Hong Kong. The Hospital Authority is a statutory body responsible for approximately 80% of chronic disease care locally.

### Participants

The inclusion criteria were primary care patients aged ≥40 years. The exclusion criteria included the inability to understand Cantonese, refusal to participate, pregnancy, having had a urinary tract infection in the past 4 weeks or being too ill to give consent.

### Sampling

Random sampling was used. To avoid disturbing service delivery and doctors’ consultations and to ensure that no patients were missed, a consultation room was randomly selected for each half-day session. Randomisation of the consultation rooms was performed prior to data collection and in discussion with the clinic staff to ensure that the room would be used for medical consultations. After randomisation, consecutively presenting patients in that consultation room were approached and recruited by a trained fieldworker. All eligible patients were invited to participate in the study.

### Study outcomes and study instruments

To measure nocturia (the independent variable), the study participants were asked to report the average number of nocturia episodes per night over a 1-month period on a 5-point Likert scale (0, 1, 2, 3 or ≥ 4 episodes per night). To make our study findings more comparable to those of previous studies [3], nocturia was defined as having ≥2 episodes per night. We used a cut-off of 2 voiding episodes per night for two reasons. First, using the International Continence Society’s definition of at least one episode per night could easily lead to a very high prevalence of nocturia [3]. Some authors also suggest that using a cut-off of 2 voiding episodes per nigh are more clinically relevant [9, 10]. Our participants were divided into 2 groups using this cut-off: (i) no nocturia (0 to 1 episode per night) and (ii) having nocturia (≥2 episodes per night).

To measure sleep quality (the proposed mediator), the Chinese version of the Pittsburgh Sleep Quality Index (PSQI) was used [11]. It is the most commonly used generic measure in both clinical and research settings, with strong positive evidence for its psychometric properties [12]. The recall period of the PSQI was 1 month. Nineteen individual items generated 7 component scores: subjective sleep quality, sleep latency, sleep duration, habitual sleep efficiency, sleep disturbances, use of sleeping medication and daytime dysfunction. The component scores ranged from 0 to 3. These 7 component scores were then summed to generate the global score of subjective sleep quality. The global score ranged from 0 to 21, with a higher score indicating poorer subjective sleep quality. A study in Hong Kong supported this unidimensional structure [13]. Additionally, a study on Chinese people suggested that a global score > 5 with a sensitivity of 98% could be used to identify people with poor sleep quality [11].

The Hong Kong Chinese version of the 12-Item Short Form Health Survey version 2 (SF-12 v2) was used to measure generic HRQOL (the dependent variable). It comprises 8 domains: physical functioning (PF), role limitation due to physical problems (RP), bodily pain (BP), general health (GH), vitality (VT), social functioning (SF), role limitation due to emotional problems (RE) and mental health (MH). The scores range from 0 to 100. The SF-12 v2 can also be summarised into 2 norm-based summary scores: physical and mental component summaries (PCS and MCS, respectively). Higher SF-12 v2 scores indicate better generic HRQOL. The SF-12 v2 has been validated and is used extensively in Hong Kong [14–16].

### Statistical analysis

First, descriptive statistics were used to describe the prevalence of symptomatic nocturia (≥2 episodes per night) and poor sleep quality (a PSQI global score > 5). Second, the association between nocturia and sleep quality was explored by multiple linear regression (using the continuous PSQI global score as the outcome) and multiple logistic regression (using the dichotomous variable: a global score ≤ 5 vs. a global score > 5). The regression models were controlled for age, gender, marital status and employment status. Third, independent *t*-tests were used to compare the SF-12 v2 score (i) between the participants with nocturia and those without and (ii) between the participants with sleep disturbance and those without. Fourth, the PROCESS macro version 3.4 developed by Hayes was used to conduct the mediation analysis [17]. In the macro, the following regression models were used:

1. HRQOL (dependent variable) was regressed on nocturia (≥2 episodes per night).
2. Sleep quality (mediators) was regressed on nocturia. We used the global score the PSQI (a continuous variable) in the analysis.
3. HRQOL was regressed on sleep quality.
4. HRQOL was regressed on both sleep quality and nocturia simultaneously.

To further validate the indirect effects (i.e. nocturia affected sleep quality, which in turn affected HRQOL), the bootstrap method was used to obtain the point estimate. This method is more powerful than the Sobel test [18]. The point estimates were based on 10,000 bootstrap samples, and 95% confidence intervals (CI) were constructed. An indirect effect was considered significant if the CI did not contain 0.

**Fig. 1:**
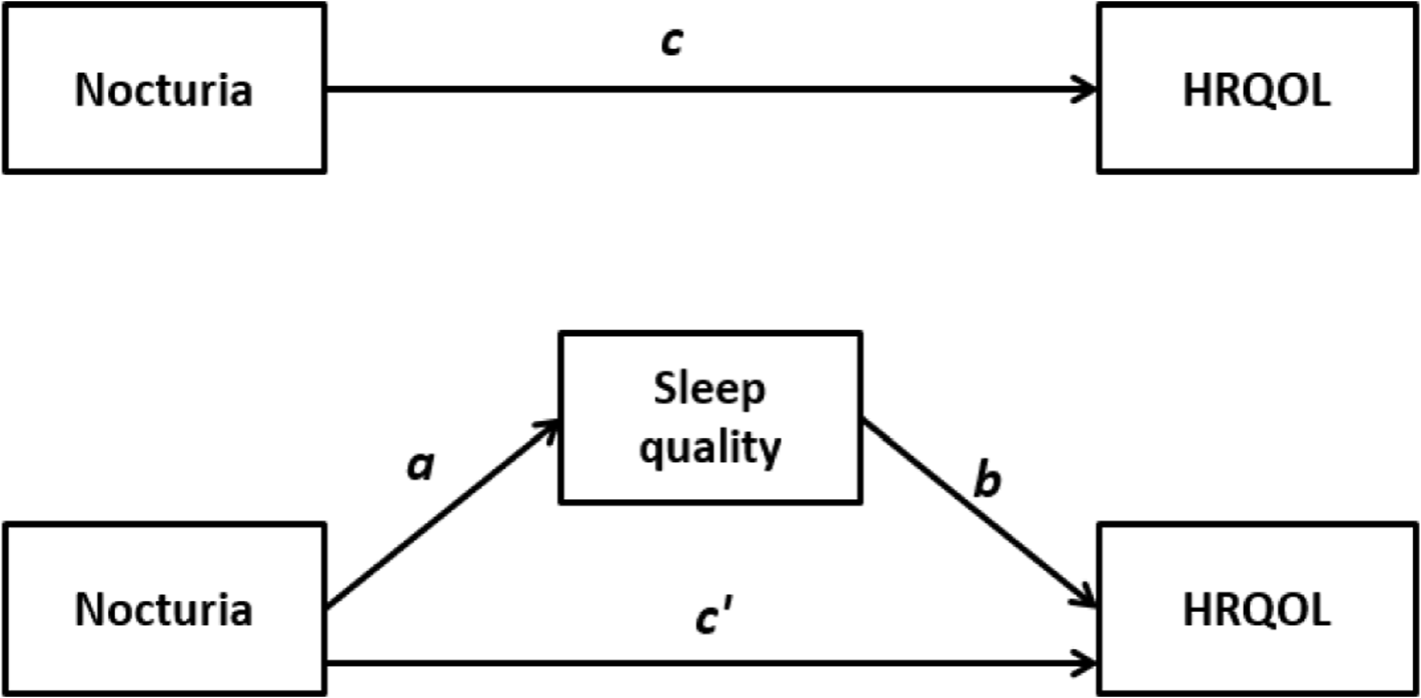
The proposed mediation model. **a** regression coefficient between nocturia and sleep disturbance. **b** regression coefficient between sleep disturbance and HRQOL. **c’** regression coefficient between nocturia and HRQOL, after controlling for sleep distance (direct effect). **c** regression coefficient between nocturia and HRQOL, without controlling for sleep distance (total effect)

Figure 1 shows the proposed mediation model. Fig. 1 shows the total effect of nocturia on HRQOL, without considering the mediator (i.e. sleep quality). *c* is the regression coefficient in a linear regression model predicting HRQOL from nocturia.

In Fig. 1, *a* is the regression coefficient in a linear regression model predicting sleep quality from nocturia whilst *b* and *c’* are the regression coefficients in a linear regression model predicting HRQOL from sleep quality and nocturia, respectively. *c’* quantifies the direct effect of nocturia on HRQOL whereas the product of *a* and *b* (point estimate) quantifies the indirect effect of nocturia on HRQOL through sleep quality. In other words, *c* = *c*’ + *ab*. The product *a* and *b* can be interpreted as the amount by which two cases who differ by one unit on nocturia are expected to differ on HRQOL though the effect of nocturia on sleep quality.

Full mediation is the case in which an independent variable no longer affects a dependent variable after a mediator has been controlled. In other words, *c*’ is no longer statistically significant. Partial mediation is the case in which the path from independent variable to dependent variable is reduced in absolute size after controlling a mediator but *c*’ is still statistically significant.

### Sample size calculation

The study by Kobelt, Borgström [5] reported that Cohen’s d effect size difference of the VT score between people with nocturia and those without was 0.67. Using this as our reference, a sample of at least 72 participants (36 in each group) was needed to detect a moderate effect size difference by independent *t*-test with 80% power (alpha = 0.05, two-tailed).

## Ethics

The study protocol was approved by the institutional review board: HKWC (UW19–179). Written informed consent was obtained from all individual participants included in the study.

## Results

### Participants

Of the 815 primary care patients who were approached, 20 patients did not meet the inclusion criteria, and 295 patients refused to join the study. As a result, 500 patients joined our study. The mean age was 57.5 years (standard deviation: 9.8), of which 57.4% were female patients. Among the participants, 74% were married, and 59.6% were in active employment. Table 1 shows the demographic characteristics of the participants.

**Table 1:** Socio-demographics and the prevalence of nocturia and poor sleep quality (*N* = 500)

*Socio-demographics*	
Mean age (SD)	57.5 (9.8)
	N (%)
Gender	
Women	287 (57.4%)
Men	213 (42.6%)
Marital status	
Not married	130 (26.0%)
Married	370 (74.0%)
Employment status	
Working	202 (40.4%)
Not working	298 (59.6%)
*Nocturia*	
≥ 1 episode per night	400 (80.0%)
≥ 2 episode per night	156 (31.2%)
≥ 3 episode per night	56 (11.2%)
*Sleep quality*	
Mean PSQI global score (SD)	7.1 (3.7)
PSQI global score > 5	302 (60.4%)
Comorbidities	
Hypertension	250 (50.0%)
Diabetes	75 (15.2%)
Heart disease	58 (11.6%)
Stroke	11 (2.2%)
Respiratory disease	30 (6.0%)
Benign prostatic hyperplasia	33 (15.5%) ^

Abbreviation; *SD* standard deviation, *PSQI* Pittsburgh Sleep Quality Index

*Note: ^ male participants only*

### Prevalence of Nocturia and sleep quality and their association

Among the participants, 80% reported having ≥ 1 nocturia episode per night, 31.2% indicated having ≥ 2 nocturia episodes per night, and 11.2% indicated that they had had ≥ 3 nocturia episodes per night. Furthermore, 60.4% of the participants had poor sleep quality. Table 1 shows the descriptive statistics of the study outcomes. Having ≥2 nocturia episodes per night increased the likelihood of having poor sleep quality, with an adjusted odds ratio (aOR) of 2.06 and β of 1.64. Table 2 shows the results of the regression analysis.

**Table 2:** regression models to explore the association between nocturia and poor sleep quality

	*Multiple logistic regression ^*			*Multiple linear regression ^^*		
	aOR	95% CI	p-value	β	95% CI	p-value
Nocturia ≥2	2.06	(1.34, 3.16)	< 0.01	1.64	(0.91, 2.38)	< 0.01
Age	0.99	(0.97, 1.01)	0.35	−0.02	(− 0.06, 0.01)	0.20
Gender	0.90	(0.62, 1.32)	0.59	−0.70	(−1.37, − 0.03)	< 0.05
Marital status	0.80	(0.52, 1.22)	0.30	−0.61	(−1.35, 0.14)	0.11
Employment status	1.24	(0.79, 1.92)	0.35	0.21	(−0.57, 0.99)	0.60
	Nagelkerke R^2^: 0.037			R^2^ 0.054		

^: the dependent variable of the model is dichotomous (PSQI global score ≤ 5 vs. 5PSQI global score > 5)

^^: the dependent variable of the model is continuous (PSQI global score)

Abbreviation: *aOR* adjusted odds ratio*, CI* confidence interval*, PSQI* Pittsburgh Sleep Quality Index*, SD* standard deviation

Compared with the patients with < 2 nocturia episodes per night, the patients with ≥2 nocturia episodes per night had poorer HRQOL as measured by the SF-12 v2 domains PF (effect size = 0.34), RP (effect size = 0.21), GH (effect size = 0.60), VT (effect size = 0.30) and SF (effect size = 0.22) and the SF-12 v2 PCS (effect size = 0.38). Compared with the patients with no sleep problems, the patients with poor sleep quality had poorer HRQOL in all 8 domains and the PCS and MCS of the SF-12 v2. The difference in effect size ranged from 0.80 for the MCS to 0.41 for the PF domain. Table 3 shows the results of the independent *t*-tests.

**Table 3:** The impacts of nocturia and poor sleep quality on health-related quality of life

	Nocturia			Sleep disturbance		
	(Nocturia < 2)	(Nocturia ≥2)	Cohen’s d Effect size	No (PSQI global score ≤ 5)	Yes (PSQI global score > 5)	Cohen’s d Effect size
	Mean (SD)	Mean (SD)		Mean (SD)	Mean (SD)	
SF-12 v2 *						
Physical Functioning	87.39 (20.80)	79.22 (27.09) **	0.34	90.31 (19.65)	81.27 (24.67) **	0.41
Role Physical	79.80 (21.70)	74.76 (25.46) *	0.21	84.85 (19.62)	73.88 (24.07) **	0.50
Bodily Pain	66.98 (27.20)	63.62 (29.28)	0.12	74.24 (26.11)	60.51 (27.70) **	0.51
General Health	54.25 (26.43)	38.68 (25.38) **	0.60	57.36 (25.84)	44.02 (26.58) **	0.51
Vitality	59.04 (23.69)	51.76 (24.61) **	0.30	66.41 (22.43)	50.42 (23.23) **	0.70
Social Functioning	81.29 (22.43)	75.96 (26.77) *	0.22	88.38 (17.20)	73.83 (26.00) **	0.66
Role Emotional	79.54 (20.91)	75.00 (25.36)	0.20	87.56 (17.11)	71.92 (23.40) **	0.76
Mental Health	69.95 (17.79)	66.27 (22.02)	0.18	76.26 (16.42)	63.91 (19.44) **	0.69
Physical Component Summary	48.81 (8.19)	45.48 (9.24) **	0.38	49.83 (7.20)	46.38 (9.27) **	0.42
Mental Component Summary	50.81 (9.48)	48.99 (11.15)	0.18	54.63 (8.02)	47.29 (10.21) **	0.80

^: independent t-test, p-value < 0.05

^^: independent t-test, p-value < 0.01

*: higher scores indicating better health-related quality of life

Abbreviation: *PSQI* Pittsburgh Sleep Quality Index*, SF-12 v2* the 12-Item Short Form Health Survey version 2*, SD* standard deviation

### Mediation models

**Fig. 2:**
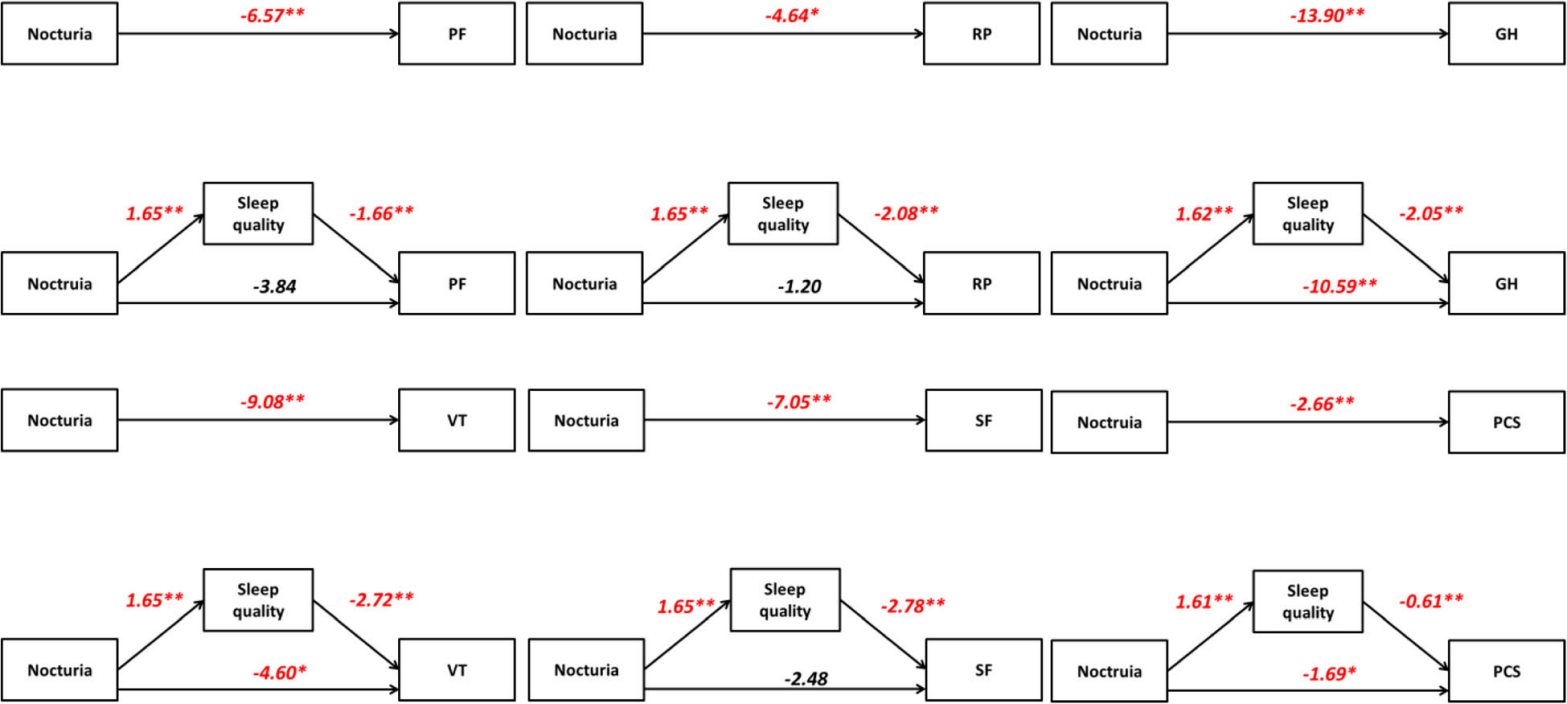
Result of the mediation analysis. * p-value <0.05, ** p-value <0.01

We only conducted the mediation analysis for the PF, RP, GH, VT and SF domains and the PCS because we only found HRQOL score differences in these domains between patients with and without ≥2 nocturia episodes per night. After controlling for sleep quality, the impacts of nocturia on HRQOL as measured by the PF (β = − 3.84, *p*-value = 0.089), RP (β = − 1.20, *p*-value = 0.593) and SF (β = − 2.48, p-value = 0.265) domains were no longer statistically significant, supporting the full mediation models. On the contrary, the direct effects of nocturia on HRQOL as measured by the GH (β = − 10.59, *p*-value < 0.01) and VT (β = − 4.60, p-value < 0.05) domains and PCS (β = − 1.69, p-value < 0.05) were still statistically significant, but the coefficients of the direct effect were smaller than those of the total effect: GH (β = − 13.90, p-value < 0.01), VT (β = − 9.08, p-value < 0.01) and PCS (β = − 2.66, p-value < 0.01), thus supporting the partial mediation models. For all the mediation models, the point estimate was negative and statistically different from 0 (i.e. the 95% CI did not contain 0). The results supported our hypothesis that sleep quality was the mediator between nocturia and HRQOL. Table 4 and Fig. 2 show the results of the mediation analysis.

**Table 4:** Mediation analysis

	Point estimate of indirect effect	95% CI ^
Physical Functioning	−2.73	−4.46, −1.27
Role Physical	−3.44	−5.50, −1.72
General Health	−3.31	−5.21, − 1.66
Vitality	− 4.48	−6.94, − 2.29
Social Functioning	− 4.57	−7.06, − 2.37
Physical Component Summary score	−0.98	− 1.65, −0.44

^: Bootstrap confidence interval: if the 95% confidence interval (CI) does not contain zero, the mediation (indirect effect) is significant. The analysis was adjusted by age, gender, marital status and employment status

Abbreviation: *CI* confidence interval

## Discussion

In our study, we found that sleep quality was a mediator in the association between nocturia and HRQOL, suggesting that nocturia affects sleep quality, which in turn affects HRQOL. One possible mechanism is that nocturia affects slow-wave sleep (SWS), which is associated with daily process of recuperation [19]. It was reported that adults who wake early to void during their first two sleep cycles spend 34% less time in SWS than adults who sleep undisturbed through two sleep cycles [20]. Moreover, disruption in sleep continuity throughout the night can also reduce SWS. Despite equivalent total sleep times, people who experience repeated awakenings during the night spend less time in SWS than people with uninterrupted sleep [20]. It was reported that interruption of SWS leads to fatigue, increased discomfort and a decreased pain threshold [21]. The findings of this mediation analysis provide additional depth to previous research. For example, the respondents in a qualitative study reported that nocturia impaired sleep in terms of quantity and quality. The next day, the respondents would feel very tired due to a lack of sleep. As a result, they could not concentrate at work [8]. An epidemiological study among a Dutch population found that the impacts of nocturia on HRQOL became statistically insignificant when sleep quality was put in a regression model [22]. No doubt, the findings that nocturia affects sleep and HRQOL are certainly not new. However, previous studies only examined the negative impacts of nocturia on sleep and HRQOL in isolation [4].

Given that nocturia itself is not life threatening, the treatment outcomes should aim to alleviate its negative impacts on daily life. Understanding the interrelationship between nocturia, sleep quality and HRQOL can therefore guide clinical practice. One important implication of our findings is that, to optimize the HRQOL of patients with nocturia, behavioural interventions for nocturia should also target sleep quality in these patients. For example, we might incorporate cognitive-behavioural theory to enhance sleep hygiene among patients suffering from nocturia.

Poor sleep quality was very common in our sample. Using the PSQI global score with > 5 as the cut-off, we found that 60% of our participants were suffering from poor sleep quality. Our prevalence was much higher than the prevalence reported in the population-based studies in Hong Kong (PSQI global score > 5: 39.4%) by Wong and Fielding [23], Germany (PSQI global score > 5: 36%) by Hinz, Glaesmer [24] and China (PSQI global score > 5: 26.6%) by Tang, Liao [25]. The high prevalence found in the present study could be explained by the characteristics of our study population. Compared with community samples, our study population in primary care were likely to include participants with multi-morbidities [26]. It was found that multi-morbidities were associated with insomnia [27].

Almost one third (31.2%) of our primary care patients were suffering from nocturia. This prevalence is similar to those reported in Korea [28], the US, the UK and Sweden [2]. These collective findings suggest that the burden of nocturia is common and universal across different populations. Moreover, the high prevalence of nocturia found in our primary care patients implies that the problems were likely overlooked and untreated.

In our study, having ≥2 nocturia episodes per night was associated with poorer HRQOL. It also appeared that nocturia had more negative impacts on the physical aspects of HRQOL than the mental aspects. However, our findings were different from those reported by Kupelian and colleagues [29]. This US study found that nocturia affected both the physical and mental aspects of HRQOL as measured by the SF-12. In fact, HRQOL and symptom perception are culturally specific. A qualitative study in the UK found that patients with urinary problems had concerns about the aetiology of their symptoms as well as disease progression. In some cases, patients even thought that their urinary problems were related to the possibility of cancer [30]. In contrast, a recent qualitative study in Hong Kong found that urinary problems had no psychological effects on the majority of Chinese patients. These patients indicated that urinary problems were just a consequence of normal ageing [31]. Even though some people reported negative psychosocial impacts associated with urinary problems, the psychological burden was merely related to embarrassment, inconvenience and restrictions on social activities [31]. These qualitative findings (i.e. urinary problems as a normal part of the ageing process and urinary problems affecting social and physical activities) by Suen and colleagues [31] echoed our findings that nocturia among the participants in our study mainly affected their physical functioning, social functioning and general health, all to the detriment of the overall physical aspects of their HRQOL.

Poor sleep quality in our study was associated with poor HRQOL across all domains of the SF-12 v2. A study of an older population in the US also found that sleep problems negatively affected all 8 domains and the PCS and MCS of the SF-36 [32]. In addition, another study found that insomnia and its associated daytime sleepiness impaired cognitive functioning such as concentration, memory, reasoning and problem solving, as well as the ability to perform ordinary daily tasks [33]. Qualitative studies have also reported that sleep problems have a pervasive impact on daily life [34], and the problems are unpleasant and worrying [35]. In our study, we found that the participants who reported sleep disturbance had poorer mental aspects of HRQOL than those without, with a large effect size. It is suggested that sleep deprivation could induce dysphoria, increase irritability and lower frustration tolerance. Experimental studies have found that sleep deprivation can lead to a deterioration in mood and increased reactions to negative emotional information [36]. Besides, based on the Cohen’s d effect sizes found in the present study, it appeared that poor sleep quality had more negative impacts on all aspects of HRQOL (except for the GH domain of the SF-12 v2) than nocturia did. For example, the effect size difference of the SF-12 v2 MCS between people with poor sleep quality than those without was 0.80 while it was 0.18 between people with nocturia than those without. The effect size difference of the SF-12 v2 PCS between people with poor sleep quality than those without was 0.42 while it was 0.38 between people with nocturia than those without. Our findings were in line with those found in Japan [37].

Our study had some limitations. First, given its cross-sectional design, causality could not be provided in the present study. Longitudinal studies are needed to make causal inferences. In fact, it is possible that people with insomnia will go to the toilet just because they can’t sleep. Our findings should be therefore interpreted with caution. Second, all the study outcomes were self-reported and may therefore be susceptible to biases such as recall bias and social desirability bias. Third, our study was conducted in public sector primary care setting. Our study findings might therefore not be transferable to the general population. Fourth, we had a high non-response rate of our study. It might lead to a non-response bias. A common reason to refuse the study was that patients did not want to spend extra time to fill out the questionnaire. It implied that waiting room screening might not be an optimal way to recruit primary care patients. Future studies should consider other strategies such as telephone follow-up interview so that patients can finish the questionnaire at their convenience.

## Conclusions

Sleep disturbance as a mediator in the association between nocturia and HRQOL suggests that nocturia not only has a direct relationship with HRQOL, but also an indirect relationship via sleep disturbance. Clinicians wanting to improve patients’ HRQOL should not only focus on the patients’ nocturia symptoms, but also on their sleep quality.

## Data Availability

The datasets used in the current study are available from the corresponding author on reasonable request.

## Abbreviations

aOR: adjusted Odds Ratio
BP: Bodily Pain
GH: General Health
HRQOL: Health-related Quality of Life
MCS: Mental Component Summaries
MH: Mental Health
OR: Odds Ratio
PCS: Physical Component Summaries
PF: Physical Functioning
PSQI: Pittsburgh Sleep Quality Index
RE: Role Limitation due to Emotional Problems
RP: Role Limitation due to Physical Problems
SF: Social Functioning
SF-12 v2: Short Form-12 Health Survey version 2
SF-36: Short Form-36 Health Survey
UK: United Kingdom
US: United States
VT: Vitality
